# Shining light on photosynthesis

**DOI:** 10.1093/jxb/eraf096

**Published:** 2025-05-16

**Authors:** Mike T Page, Martin A J Parry

**Affiliations:** Journal of Experimental Botany, Lancaster, UK; Lancaster Environment Centre, Lancaster University, Lancaster, UK

**Photosynthesis is the primary process by which plants harness the energy of sunlight to convert water and carbon dioxide into energy-rich organic compounds. These compounds serve as the foundation for all food chains on the planet. Moreover, photosynthesis plays a vital role in the global carbon cycle, as photosynthetic organisms help regulate the Earth’s climate by sequestering carbon and reducing greenhouse gas concentrations. Photosynthesis is a complex trait determined by numerous component traits that are themselves multigenic. Understanding the intricacies of photosynthesis is crucial in developing strategies to improve crop productivity, support sustainable agricultural practices, and mitigate the impacts of climate change.**

The *Journal of Experimental Botany* (*JXB*) has a long history of publishing impactful research in this field, evident even in the first issue of the journal in March 1950 with an advance in understanding the ‘path of carbon in photosynthesis’ by Andrew Benson and Melvin Calvin (Benson and Calvin, 1950) (Fig. 1). The journal continues to publish high-quality and important photosynthesis research, and this Virtual Issue focuses on recent progress in revealing the intricacies of photosynthesis. It brings together a collection of manuscripts that exemplify innovative advances in technology and research, showcasing our understanding of the complex mechanisms, adaptations, and environmental influences that govern photosynthesis.

**Fig. 1.**
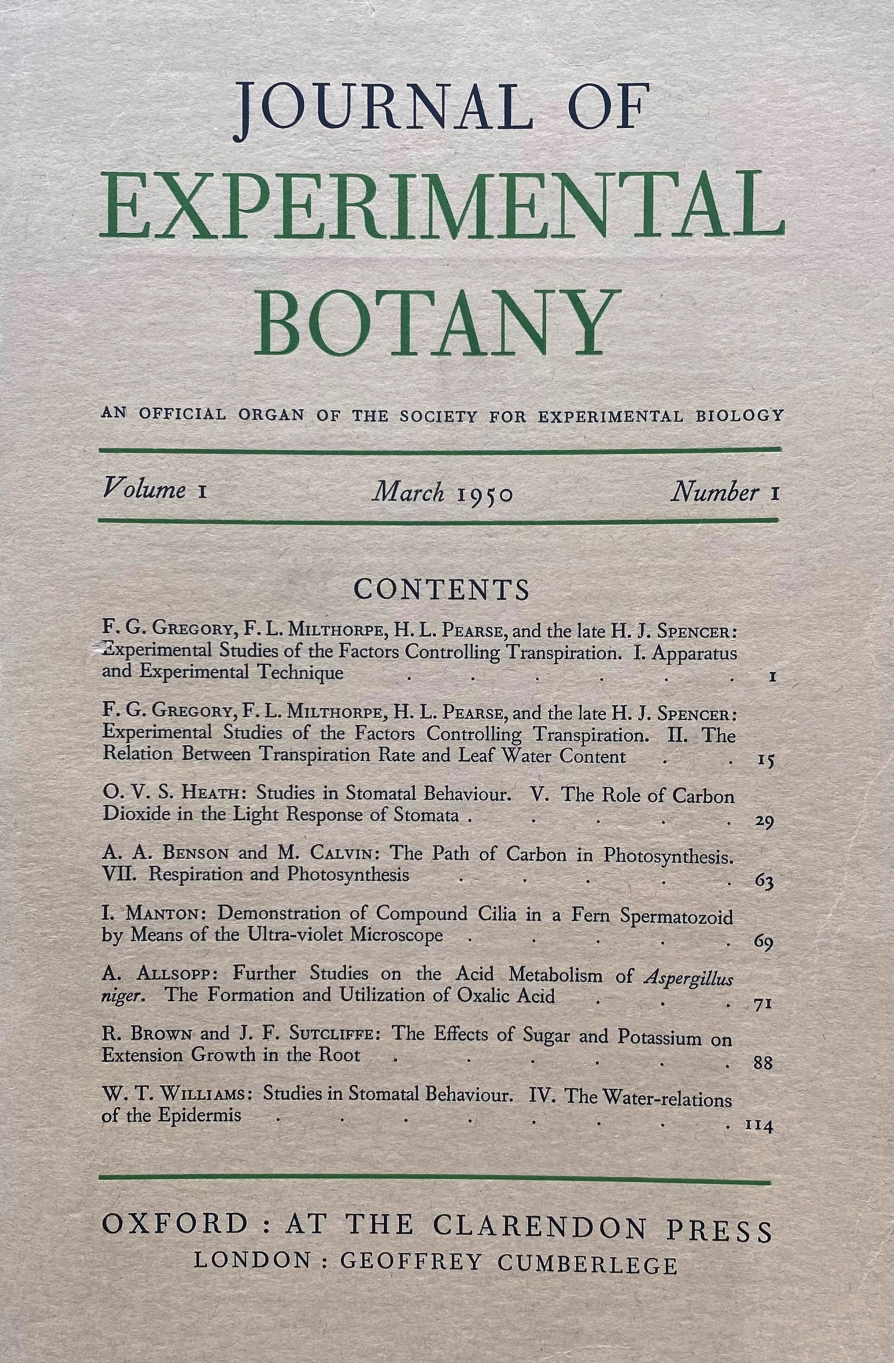
A photograph of the front cover of the first issue of the *Journal of Experimental Botany*, published in March 1950.

## Method development

In 2020, *JXB* introduced a new type of paper dedicated to the communication of new and improved research methods. The first of these Technical Innovations was an analysis of plate-based methods for measuring Rubisco activity (Sales *et al*., 2020). Given the importance of Rubisco in photosynthesis, the provision of cost-effective, reliable, and simple alternatives to the traditional radiometric assays is vital to the photosynthetic research effort. Since 2020, the journal has published many more method advances related to photosynthesis. Some of these have sought to update well-established methods, for example: a revision to the steady-state model of C_4_ photosynthesis (von Caemmerer, 2021); a correction to the PSI quantum yield equation, demonstrating that the previously reported seasonal up-regulation of cyclic electron transfer (CET) during spring recovery of photosynthesis in boreal conifers is in fact absent (Grebe *et al*., 2024); and modification of the ‘one-point method’ enabling accurate estimation of the maximum carboxylation rate of Rubisco at higher temperatures (Oliveira *et al*., 2023). Other methods have improved the efficiency of photosynthesis research, for example through the continued development of an *Escherichia coli*-based expression system for testing heterologous Rubisco production *in planta* (Buck *et al*., 2023), and the demonstration that the Dynamic Assimilation Technique (DAT) can reduce the measurement time of *A*/*C*_i_ curves by almost a half without loss of accuracy compared with conventional steady-state methods (Tejera-Nieves *et al*., 2024). Ferguson *et al*. (2023) established differences in measured photosynthetic traits between excised and attached leaves across three crop species, an important observation for photosynthesis researchers performing similar experiments. Embracing an exciting and developing technology, two groups independently reviewed the use of artificial intelligence and machine learning for rapid and robust stomatal phenotyping, highlighting the current capabilities, limitations, and future directions for the technology (Gibbs and Burgess, 2024; Tan *et al*., 2024), while another group used machine learning to predict Rubisco kinetics (Iqbal *et al*., 2023).

## Exploring and exploiting genetic variation

Characterizing the genetic diversity of photosynthetic traits, both within and between species, can help us to better understand photosynthetic mechanisms. It also provides a valuable resource to identify potential targets to modulate aspects of photosynthesis for various applications. In their study, Gao *et al.* (2024) screened 23 field-grown inbred barley lines by measuring chlorophyll fluorescence and growth traits, identifying genetic variation in photosynthetic parameters that correlated with yield. The development of a high-throughput screening method was also used in identifying exploitable genetic variation in bread wheat (Faralli *et al*., 2024). In this case, combining gas exchange and thermal imaging revealed significant variation in photosynthetic and stomatal traits among bread wheat genotypes, including CO_2_ uptake rates, stomatal dynamics, and evaporative cooling. Another impressive example is the high-throughput phenotyping of African and Asian rice accessions for photosynthetic heat tolerance, focusing primarily on the heat-sensitive PSII complex followed by genome-wide association studies (Robson *et al*., 2023). This approach identified 133 candidate genes for improving photosynthesis under high temperature, paving the way for breeding heat-tolerant rice varieties. Using both glasshouse and field phenotyping, an analysis of 178 climbing bean lines was also successful in exploring variation in photosynthetic efficiency to identify a potential target to boost yield (Keller *et al*., 2024).

## Coping with fluctuating light

Fluctuating light can disrupt the balance of ATP and NADPH production and consumption in photosynthesis, leading to damaging oxidative stress. A symptom of such conditions is the oscillation of CO_2_ assimilation and fluorescence parameters. Impairment of Proton Gradient Regulation 5 (PGR5)-dependent CET heightened these oscillations, identifying a major role for this protein in dampening photosynthetic stress during environmental fluctuations (Degen *et al.*, 2024). Employing DATs, further research into the underlying mechanics of such oscillations provided crucial insight into how they respond to triose phosphate utilization (TPU) and changes in CO_2_ concentration, deepening our understanding of the dynamic behaviour of photosynthesis (McClain and Sharkey, 2023). When excess light is absorbed by the photosynthetic machinery, plants may experience photoinhibition if photoprotective systems cannot repair the damage rapidly enough. To date, the majority of research into photoinhibition has been conducted in photosynthetic organisms that are not particularly tolerant to high light. Levin and Schuster (2024) argue eloquently that photoprotection should be studied predominantly in high-light-tolerant species to better identify and characterize efficient protective mechanisms.

As well as studying the response of photosynthesis to changing light conditions at the organellar scale, significant advances have been made in recent years in our knowledge of how photosynthesis responds at the canopy scale (reviewed in Sellaro *et al*., 2025). A recent example of this is a study of leaves from different positions in mature tomato canopies (Shao *et al*., 2024). Leaves at the top of the canopy were faster to adjust photosynthesis and photoprotective systems in response to changing light than leaves from lower in the canopy. A model analysis of the canopy-level data revealed that a single set of parameters was sufficient to predict whole-canopy assimilation under fluctuating light.

## Photosynthesis in a changing climate

The threat of climate change looks set to present future crops with higher atmospheric CO_2_ concentrations, warmer temperatures, and unpredictable weather events. Consequently, considerable research effort is invested in understanding how plants might fare under such conditions, especially in response to high temperature stress (reviewed by Moore *et al*., 2021; Qu *et al*., 2023). In Arabidopsis, Cavanagh *et al.* demonstrate that differential expression of Rubisco small subunits in response to high temperature produces Rubisco protein with an enhanced carboxylation capacity, sufficient to compensate for a concomitant reduction in Rubisco content at higher temperatures (Cavanagh *et al*., 2023). Another study looked at the photosynthetic responses of wheat genotypes to warm nights, and found that night-time warming directly impacted wheat photosynthesis and its acclimation ability, with distinct responses between newly formed and pre-existing leaves (Coast *et al*., 2024). Al-Salman *et al.* (2023) looked at the combined effects of elevated CO_2_ (eCO_2_), high temperature, and water stress on photosynthesis in the C_4_ crop sorghum, revealing leaf stomatal conductance as the central player in a trade-off between improving water use efficiency and maintaining adequate thermoregulation. The intersection of eCO_2_ and unfavourable climatic conditions was further probed by Ancín *et al*. (2024) who showed that photosynthesis is boosted by eCO_2_ in C_3_ and C_4_ plants, although the carboxylation rate and Rubisco activity were reduced in C_3_ species. These responses were largely unmodified by drought or heat stress, providing further insight into how photosynthesis may be affected by a changing climate.

## Engineering improved photosynthesis

The majority of plants employ C_3_ photosynthesis to fix carbon, but other types of photosynthesis have evolved that concentrate CO_2_ around Rubisco to inhibit its oxygenation reaction and increase carboxylation. These include biochemical carbon-concentrating mechanisms (CCMs) such as C_4_ and Crassulacean acid metabolism (CAM) photosynthesis, as well as biophysical CCMs such as the cyanobacterial carboxysome and the algal pyrenoid. Research to advance our understanding of these other forms of photosynthesis may hold the key for engineering C_3_ crops with improved photosynthetic efficiency. Schlüter *et al.* (2023) performed a phylogenetic analysis of the C_3_–C_4_ intermediate phenotype in *Brassicaceae* species, revealing that it has evolved up to five times independently in this plant family, with varying efficiency. The convergent evolution of this feature suggests that it is beneficial under certain environmental conditions and may be a good target for transfer to C_3_*Brassicaceae* crops to improve their metabolic plasticity under a dynamic climate. Another study used a 3D maize leaf model to predict how chloroplast movement might affect C_4_ photosynthesis (Retta *et al*., 2023). The movement of chloroplasts towards mesophyll cells increased light absorption and photosynthetic rates, while the converse was true for chloroplast avoidance movement (away from bundle sheath cells), an important insight when attempting to engineer C_4_ photosynthesis into C_3_ crops. Staying in maize, Eshenour *et al*. (2024) further probed the opportunities for boosting C_4_ photosynthesis through increased expression of Rubisco subunits and assembly factors.

Turning attention to the biophysical CCMs, the *Chlamydomonas* LCIA protein has long been considered a membrane bicarbonate transporter in the algal CCM. This was finally demonstrated for the first time by expressing LCIA in mutant bacterial and plant systems which are normally unable to grow in ambient CO_2_ (Förster *et al*., 2023). LCIA expression rescued growth in both systems, indicating that LCIA was enabling bicarbonate uptake, confirming it as a crucial component of the algal CCM. The BCT1 bicarbonate transporter is a complex of the cyanobacterial CCM that holds promise for engineering improved photosynthetic efficiency in C_3_ plants. However, correct targeting of the four components to the correct chloroplast locations is extremely challenging. A rational design and directed evolution approach yielded constitutively active versions of BCT1, although none was able to boost photosynthesis *in planta* (Rottet *et al*., 2024). However, this type of modification of CCM components does represent a viable strategy for improving plant photosynthesis.

## Recent *JXB* special issues related to photosynthesis:

Rubisco and its Regulation (2023): https://academic.oup.com/jxb/issue/74/2. Edited by Elizabete Carmo-Silva and Rob Sharwood.

Stomata—Gatekeepers to Future Plant Performance (2024): https://academic.oup.com/jxb/issue/75/21. Edited by Andrew Leakey and Tracy Lawson.

The exploration of photosynthesis continues to unfold, shaping our understanding and our ability to tackle global challenges. This *JXB* Virtual Issue serves as a platform to celebrate the exciting progress made in both unravelling its complexities and translating that knowledge to address global challenges. We hope the research described here can inspire further exploration, collaboration, and innovation in photosynthesis research to unlock the potential of this fundamental process and chart a path towards a greener and more sustainable world.

## References

[CIT0001] Al-Salman Y , Ghannoum O, Cano FJ. 2023. Elevated [CO_2_] negatively impacts C_4_ photosynthesis under heat and water stress without penalizing biomass. Journal of Experimental Botany 74, 2875–2890. 36800252 10.1093/jxb/erad063 PMC10401618

[CIT0002] Ancín M , Gámez AL, Jauregui I, Galmes J, Sharwood RE, Erice G, Ainsworth EA, Tissue DT, Sanz-Sáez A, Aranjuelo I. 2024. Does the response of Rubisco and photosynthesis to elevated [CO_2_] change with unfavourable environmental conditions? Journal of Experimental Botany 75, 7351–7364. 39264212 10.1093/jxb/erae379 PMC11629997

[CIT0003] Benson AA , Calvin M. 1950. The path of carbon in photosynthesis: VII. Respiration and photosynthesis. Journal of Experimental Botany 1, 63–68.

[CIT0004] Buck S , Rhodes T, Gionfriddo M, Skinner T, Yuan D, Birch R, Kapralov MV, Whitney SM. 2023. *Escherichia coli* expressing chloroplast chaperones as a proxy to test heterologous Rubisco production in leaves. Journal of Experimental Botany 74, 664–676. 36322613 10.1093/jxb/erac435

[CIT0005] Cavanagh AP , Slattery R, Kubien DS. 2023. Temperature-induced changes in Arabidopsis Rubisco activity and isoform expression. Journal of Experimental Botany 74, 651–663. 36124740 10.1093/jxb/erac379 PMC9833042

[CIT0006] Coast O , Scafaro AP, Bramley H, Taylor NL, Atkin OK. 2024. Photosynthesis in newly developed leaves of heat-tolerant wheat acclimates to long-term nocturnal warming. Journal of Experimental Botany 75, 962–978. 37935881 10.1093/jxb/erad437 PMC10837020

[CIT0007] Degen GE , Pastorelli F, Johnson MP. 2024. Proton Gradient Regulation 5 is required to avoid photosynthetic oscillations during light transitions. Journal of Experimental Botany 75, 947–961. 37891008 10.1093/jxb/erad428

[CIT0008] Eshenour K , Hotto A, Michel EJS, Oh ZG, Stern DB. 2024. Transgenic expression of Rubisco accumulation factor2 and Rubisco subunits increases photosynthesis and growth in maize. Journal of Experimental Botany 75, 4024–4037. 38696303 10.1093/jxb/erae186

[CIT0009] Faralli M , Mellers G, Wall S, et al 2024. Exploring natural genetic diversity in a bread wheat multi-founder population: dual imaging of photosynthesis and stomatal kinetics. Journal of Experimental Botany 75, 6733–6747. 38795361 10.1093/jxb/erae233 PMC11565207

[CIT0010] Ferguson JN , Jithesh T, Lawson T, Kromdijk J. 2023. Excised leaves show limited and species-specific effects on photosynthetic parameters across crop functional types. Journal of Experimental Botany 74, 6662–6676. 37565685 10.1093/jxb/erad319 PMC10662226

[CIT0011] Förster B , Rourke LM, Weerasooriya HN, et al 2023. The *Chlamydomonas reinhardtii* chloroplast envelope protein LCIA transports bicarbonate *in planta*. Journal of Experimental Botany 74, 3651–3666. 36987927 10.1093/jxb/erad116

[CIT0012] Gao Y , Stein M, Oshana L, Zhao W, Matsubara S, Stich B. 2024. Exploring natural genetic variation in photosynthesis-related traits of barley in the field. Journal of Experimental Botany 75, 4904–4925. 38700102 10.1093/jxb/erae198 PMC11523619

[CIT0013] Gibbs JA , Burgess AJ. 2024. Application of deep learning for the analysis of stomata: a review of current methods and future directions. Journal of Experimental Botany 75, 6704–6718. 38716775 10.1093/jxb/erae207 PMC11565211

[CIT0014] Grebe S , Porcar-Castell A, Riikonen A, Paakkarinen V, Aro E-M. 2024. Accounting for photosystem I photoinhibition sheds new light on seasonal acclimation strategies of boreal conifers. Journal of Experimental Botany 75, 3973–3992. 38572950 10.1093/jxb/erae145 PMC11233416

[CIT0015] Iqbal WA , Lisitsa A, Kapralov MV. 2023. Predicting plant Rubisco kinetics from RbcL sequence data using machine learning. Journal of Experimental Botany 74, 638–650. 36094849 10.1093/jxb/erac368 PMC9833099

[CIT0016] Keller B , Soto J, Steier A, Portilla-Benavides AE, Raatz B, Studer B, Walter A, Muller O, Urban MO. 2024. Linking photosynthesis and yield reveals a strategy to improve light use efficiency in a climbing bean breeding population. Journal of Experimental Botany 75, 901–916. 37878015 10.1093/jxb/erad416 PMC10837016

[CIT0017] Levin G , Schuster G. 2024. Light tolerance in light-tolerant photosynthetic organisms: a knowledge gap. Journal of Experimental Botany 75, 6199–6202. 39101403 10.1093/jxb/erae338 PMC11522983

[CIT0018] McClain AM , Sharkey TD. 2023. Rapid CO_2_ changes cause oscillations in photosynthesis that implicate PSI acceptor-side limitations. Journal of Experimental Botany 74, 3163–3173. 36883576 10.1093/jxb/erad084 PMC10199117

[CIT0019] Moore CE , Meacham-Hensold K, Lemonnier P, Slattery RA, Benjamin C, Bernacchi CJ, Lawson T, Cavanagh AP. 2021. The effect of increasing temperature on crop photosynthesis: from enzymes to ecosystems. Journal of Experimental Botany 72, 2822–2844. 33619527 10.1093/jxb/erab090 PMC8023210

[CIT0020] Oliveira TCD , Garcia MN, Veenendaal E, Domingue TF. 2023. Extending the ‘one-point method’ for estimations of leaf photosynthetic capacity to a broader temperature range. Journal of Experimental Botany 74, 684–687. 36434789 10.1093/jxb/erac466 PMC9899412

[CIT0021] Qu Y , Mueller-Cajar O, Yamori W. 2023. Improving plant heat tolerance through modification of Rubisco activase in C_3_ plants to secure crop yield and food security in a future warming world. Journal of Experimental Botany 74, 591–599. 35981868 10.1093/jxb/erac340

[CIT0022] Retta MA , Yin X, Ho QT. et al 2023. The role of chloroplast movement in C_4_ photosynthesis: a theoretical analysis using a three-dimensional reaction–diffusion model for maize. Journal of Experimental Botany 74, 4125–4142. 37083863 10.1093/jxb/erad138 PMC10400148

[CIT0023] Robson JK , Ferguson JN, McAusland L, et al 2023. Chlorophyll fluorescence-based high-throughput phenotyping facilitates the genetic dissection of photosynthetic heat tolerance in African (*Oryza glaberrima*) and Asian (*Oryza sativa*) rice. Journal of Experimental Botany 74, 5181–5197. 37347829 10.1093/jxb/erad239 PMC10498015

[CIT0024] Rottet S , Rourke LM, Pabuayon ICM. et al 2024. Engineering the cyanobacterial ATP-driven BCT1 bicarbonate transporter for functional targeting to C_3_ plant chloroplasts. Journal of Experimental Botany 75, 4926–4943. 38776254 10.1093/jxb/erae234 PMC11349869

[CIT0025] Sales CRG , da Silva AB, Carmo-Silva E. 2020. Measuring Rubisco activity: challenges and opportunities of NADH-linked microtiter plate-based and ^14^C-based assays. Journal of Experimental Botany 71, 5302–5312. 32728715 10.1093/jxb/eraa289 PMC7501812

[CIT0026] Schlüter U , Bouvier JW, Guerreiro R, Malisic M, Kontny C, Westhoff P, Stich B, Weber APM. 2023. *Brassicaceae* display variation in efficiency of photorespiratory carbon-recapturing mechanisms. Journal of Experimental Botany 74, 6631–6649. 37392176 10.1093/jxb/erad250 PMC10662225

[CIT0027] Sellaro R , Durand M, Aphalo PJ, Casal JJ. 2025. Making the most of canopy light: shade avoidance under a fluctuating spectrum and irradiance. Journal of Experimental Botany 76, 712–729. 39101508 10.1093/jxb/erae334 PMC11805590

[CIT0028] Shao B , Zhang Y, Vincenzi E, Berman S, Vialet-Chabrand S, Marcelis LFM, Li T, Kaiser E. 2024. Photosynthesis and photoprotection in top leaves respond faster to irradiance fluctuations than bottom leaves in a tomato canopy. Journal of Experimental Botany 75, 7217–7236. 39171726 10.1093/jxb/erae357 PMC11630027

[CIT0029] Tan GD , Chaudhuri U, Varela S, Ahuja N, Leakey ADB. 2024. Machine learning-enabled computer vision for plant phenotyping: a primer on AI/ML and a case study on stomatal patterning. Journal of Experimental Botany 75, 6683–6703. 39363775 10.1093/jxb/erae395 PMC11565210

[CIT0030] Tejera-Nieves M , Seong DY, Reist L, Walker BJ. 2024. The Dynamic Assimilation Technique measures photosynthetic CO_2_ response curves with similar fidelity to steady-state approaches in half the time. Journal of Experimental Botany 75, 2819–2828. 38366564 10.1093/jxb/erae057 PMC11103103

[CIT0031] von Caemmerer S. 2021. Updating the steady-state model of C_4_ photosynthesis. Journal of Experimental Botany 72, 6003–6017. 34173821 10.1093/jxb/erab266 PMC8411607

